# SiO_2_ and TiO_2_ nanoparticles synergistically trigger macrophage inflammatory responses

**DOI:** 10.1186/s12989-017-0192-6

**Published:** 2017-04-11

**Authors:** Misato Tsugita, Nobuyuki Morimoto, Masafumi Nakayama

**Affiliations:** 1 Frontier Research Institute for Interdisciplinary Sciences, Tohoku University, 6-3 Aramaki-Aoba, Aoba-ku Sendai, 980-8578 Japan; 2 Department of Materials Processing, Graduate School of Engineering, Tohoku University, Sendai, 980-8579 Japan

**Keywords:** SiO_2_, TiO_2_, Nanoparticle, Macrophage, IL-1β, Inflammation

## Abstract

Silicon dioxide (SiO_2_) nanoparticles (NPs) and titanium dioxide (TiO_2_) NPs are the most widely used inorganic nanomaterials. Although the individual toxicities of SiO_2_ and TiO_2_ NPs have been extensively studied, the combined toxicity of these NPs is much less understood. In this study, we observed unexpected and drastic activation of the caspase-1 inflammasome and production of IL-1β in mouse bone marrow-derived macrophages stimulated simultaneously with SiO_2_ and TiO_2_ NPs at concentrations at which these NPs individually do not cause macrophage activation. Consistent with this, marked lung inflammation was observed in mice treated intratracheally with both SiO_2_ and TiO_2_ NPs. In macrophages, SiO_2_ NPs localized in lysosomes and TiO_2_ NPs did not; while only TiO_2_ NPs produced ROS, suggesting that these NPs induce distinct cellular damage leading to caspase-1 inflammasome activation. Intriguingly, dynamic light scattering measurements revealed that, although individual SiO_2_ and TiO_2_ NPs immediately aggregated to be micrometer size, the mixture of these NPs formed a stable and relatively monodisperse complex with a size of ~250 nm in the presence of divalent cations. Taken together, these results suggest that SiO_2_ and TiO_2_ NPs synergistically induce macrophage inflammatory responses and subsequent lung inflammation. Thus, we propose that it is important to assess the synergistic toxicity of various combinations of nanomaterials.

**Electronic supplementary material:**

The online version of this article (doi:10.1186/s12989-017-0192-6) contains supplementary material, which is available to authorized users.

## Background

With the development of nanotechnology, the production and distribution of engineered nanomaterials (ENMs) is rapidly expanding [1, 2]. The most frequently used nanomaterials are inorganic nanoparticles (NPs) such as silicon dioxide (SiO_2_) and titanium dioxide (TiO_2_) [2]. Indeed, these NPs are currently used in a wide variety of materials including paints, cosmetics, and pharmaceutical products [3, 4]. Both SiO_2_ and TiO_2_ had been considered to be biocompatible; however, numerous recent studies have shown that particle size impacts toxicity [5, 6]. For instance, while micro-sized SiO_2_ and TiO_2_ particles rarely cause inflammation, their NPs do [7–9]. Given the current expanding use of ENMs, the assessment of any health risks associated with these materials is the globally important. In this context, the toxicity of individual ENMs has been extensively studied; however, the combined toxicity of multiple ENMs has not. Therefore, because it is likely that our bodies are exposed simultaneously to a wide variety type of ENMs, the combined toxicity of multiple ENMs should be extensively addressed.

When NPs enter intentionally or accidentally into our bodies, they can be recognized and internalized by professional phagocytes such as macrophages. Some NPs, such as SiO_2_, strongly activate macrophages to induce IL-1β secretion [8, 9]. In addition, animal studies have shown that pulmonary exposure to SiO_2_ NPs causes severe inflammation [8, 9]. Furthermore, pulmonary inflammation was ameliorated by genetic deletion of IL-1β [10], suggesting that IL-1β is crucial for NP toxicity. It is thought that macrophage-secreted IL-1β binds to IL-1 receptor 1 (IL-1R1) on pulmonary epithelial cells and fibroblasts [11]. The Activated IL-1R1 then associates with the cytoplasmic adaptor protein MyD88 and transmits signals leading to activation of the transcription factor NF-κB, which induces expression of various inflammatory mediators including TNF-α, KC, and IL-6, resulting in lung inflammation [11].

Given that IL-1β is a strong pro-inflammatory mediator, secretion of IL-1β is tightly controlled and requires at least two specific signals [12, 13]. The first is mediated by pathogen-associated molecular patterns (PAMPs) such as lipopolysaccharide (LPS) and lipoproteins. These PAMPs stimulate Toll-like receptors to activate NF-κB, leading to production of pro-IL-1β along with Nod-like receptor protein (NLRP) 3. The second signal causes activation of the inflammasome, an NLRP3-containing multiprotein complex leading to activation of caspase-1, which subsequently processes pro-IL-1β into mature IL-1β. Upon internalized by macrophages, SiO_2_ NPs activate the second signal but not the first signal [8]. Further, NLRP3-deficient macrophages and mice do not secrete IL-1β in response to ENMs [14], suggesting that inflammasome activation is crucial for ENM toxicity.

We have recently reported that SiO_2_ NPs strongly induce caspase-1 inflammasome activation and subsequent pulmonary inflammation in mice [9]. Here we extended this study to address whether individual or combined inorganic NPs induce inflammation. Unexpectedly, we found that when co-administered, relatively low concentrations/doses of SiO_2_ and TiO_2_ NPs are able to synergistically activate macrophages to induce IL-1β secretion and subsequent pulmonary inflammation in mice.

## Results

### Synergistic inflammation is induced by SiO_2_ and TiO_2_ NPs

We first addressed whether individual or combined inorganic (SiO_2_, TiO_2_, NiO, Al_2_O_3_, ZnO, and Ag) NPs induce IL-1β secretion from LPS-primed B6 mouse bone marrow-derived macrophages (BMDMs). It has been reported that SiO_2_ particles rapidly activate NLRP3 inflammasomes and induce IL-1β secretion from macrophages at 3–6 h after particle stimulation [15–17]. Thus, we measured IL-1β in the culture supernatants at 4 h after NP stimulation. Consistent with our previous report [9], 100 μg/cm^3^ of SiO_2_ NPs induced a high amount of IL-1β secretion from BMDMs (Additional file 1: Figure S1a), while 10 μg/cm^3^ of SiO_2_ NPs did not (Additional file 1: Figure S1a). On the other hand, 100 μg/cm^3^ of TiO_2_ or NiO NPs only weakly induced IL-1β secretion (less than 100 pg/ml) (Additional file 1: Figure S1a) and 10 μg/cm^3^ of these NPs had no effect (Additional file 1: Figure S1a). Moreover, Al_2_O_3_, ZnO, and Ag NPs did not induce IL-1β secretion at all even at 100 μg/cm^3^ (Additional file 1: Figure S1a).

**Fig. 1:**
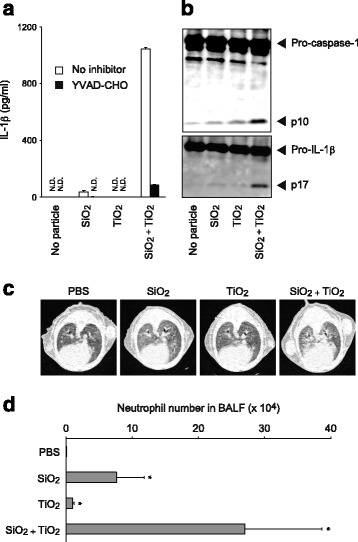
Synergistic inflammation by SiO_2_ and TiO_2_ nanoparticles (NPs). **a** LPS-primed C57BL/6 mouse bone marrow-derived macrophages (BMDMs) were untreated (white columns) or pretreated with YVAD-CHO (20 μM: black columns) and were stimulated with SiO_2_ NPs and/or TiO_2_ NPs (10 μg/cm^3^ each) for 4 h at 37 °C. The amount of IL-1β in culture supernatants was measured by ELISA. Data are shown as mean + S.D. N.D.; not detected. **b** LPS-primed BMDMs were unstimulated or stimulated with SiO_2_ NPs and/or TiO_2_ NPs (10 μg/cm^3^) for 2 h at 37 °C. Maturation of caspase-1 and IL-1β in pooled supernatants and cell extracts were analyzed by immunoblot. **c**, **d** C57BL/6 mice were intratracheally treated with PBS alone or with SiO_2_ NPs and/or TiO_2_ NPs (5 mg/kg each) (*N* = 4 per group). Twenty-four h after injection, lung inflammation was analyzed by micro-computed tomography in **c**. Bronchoalveolar lavage fluid (BALF) was harvested from these mice, and total cell number in BALF was counted. Then cells were stained with fluorescently-labeled anti-Gr-1 mAb and analyzed by flow cytometry. Gr-1-positive cell number in BALF was calculated and indicated as the mean + S.D. in **d**. Analysis of variance (ANOVA) *F*_(3,12)_ = 12.8, *P* < 0.01 for treatment groups. *P < 0.05 to the others, Holm’s *post hoc* test. Similar results were obtained in three independent studies

Intriguingly, simultaneous stimulation with SiO_2_ and TiO_2_ NPs (10 μg/cm^3^ each) synergistically induced IL-1β secretion although various combinations of other NPs did not have such an effect (Fig. 1a and Additional file 1: Figure S1b). The concentration-response study showed that 5–20 μg/cm^3^ of TiO_2_ NPs enhanced IL-1β secretion in response to 10 μg/cm^3^ of SiO_2_ NPs; however, 20 μg/cm^3^ of SiO_2_ NPs *per se* strongly induced IL-1β secretion, which was not further enhanced by TiO_2_ NPs (Additional file 1: Figure S2).

It is noteworthy that SiO_2_ and TiO_2_ NPs are the most frequently used ENMs [2]. Therefore, we further addressed this synergistic response. Because the ELISA system cannot distinguish between pro-IL-1β and mature IL-1β in culture supernatants, we used western blotting to examine inflammasome signaling. As expected, both caspase-1 and IL-1β processing were markedly induced by the combination of SiO_2_ and TiO_2_ NPs, as indicated by the increased intensity of the active caspase-1 p10 form and the mature IL-1β p17 form, respectively (Fig. 1b). This suggests that SiO_2_ and TiO_2_ NPs work synergistically to induce caspase-1 inflammasome activation leading to IL-1β processing and secretion. Indeed, IL-1β secretion was markedly inhibited by the caspase-1 inhibitor, YVAD-CHO (Fig. 1a).

It has been reported that pulmonary exposure to SiO_2_ causes acute pulmonary inflammation in mice, which reaches a maximum at 24 h after the exposure [18–20], and that the pulmonary inflammation is mediated via caspase-1 inflammasome activation [8, 9]. Thus, we utilized this mouse model to perform an in vivo study. While LPS priming is required for particle-induced IL-1β secretion in BMDMs, it is not required in mouse lungs [8, 9], suggesting that pro-IL-1β exists in the lungs of mice housed under specific-pathogen free conditions. We previously observed that a high dose (25 mg/kg) of SiO_2_ NPs can cause severe pulmonary inflammation [9]. Here, we addressed whether a low dose of SiO_2_ and TiO_2_ NPs could induce acute pulmonary inflammation at 24 h after the exposure. Although 2.5 mg/kg of SiO_2_ or TiO_2_ NPs alone did not cause pulmonary inflammation, cotreatment with this dose of NPs caused inflammation, as indicated by high intensity signals in the computed tomography (CT) images (Additional file 1: Figure S3a). Moreover, synergistic induction of lung inflammation was obvious at 5 mg/kg of NPs (Fig. 1c and Additional file 1: Figure S3). Consistent with this, massive neutrophil infiltration was observed in bronchoalveolar lavage fluid (BALF) from mice treated simultaneously with these doses of SiO_2_ and TiO_2_ NPs (Fig. 1d and Additional file 1: Figure S3b). However, at 10 mg/kg, SiO_2_, but not TiO_2_, alone caused severe inflammation, and cotreatment with this dose of these NPs did not have the synergistic effect (Additional file 1: Figure S3). Taken together, these results suggest that low doses/concentrations of SiO_2_ and TiO_2_ NPs synergistically induce inflammation in vivo as well as in vitro.

### Distinct localization of SiO_2_ and TiO_2_ NPs in macrophages

**Fig. 2:**
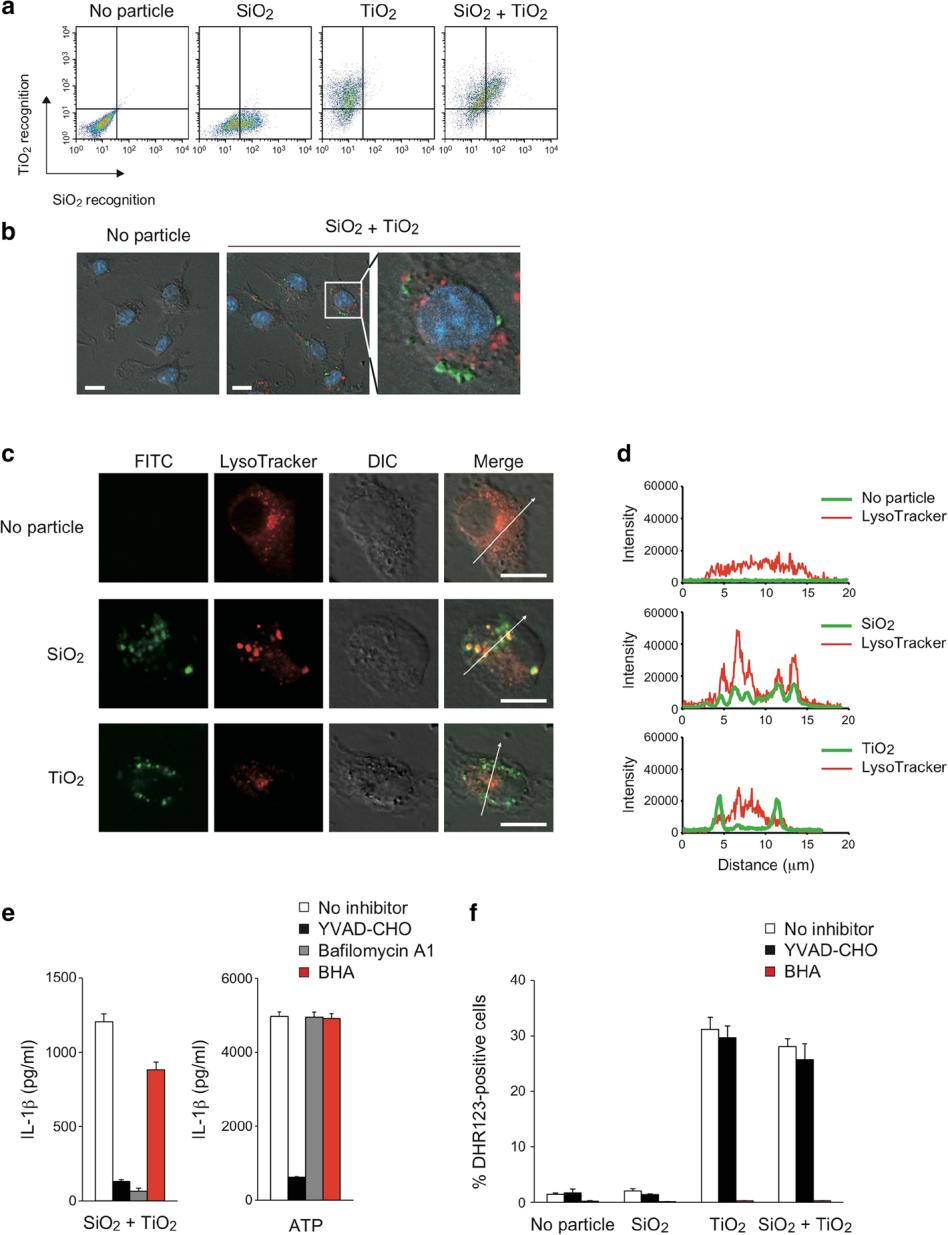
SiO_2_ and TiO_2_ NPs induce different cellular stress pathways in BMDMs. **a** LPS-primed BMDMs were stimulated with rhodamine-labeled SiO_2_ NPs and/or FITC-labeled TiO_2_ NPs (10 μg/cm^3^ each) for 30 min at 37 °C. Cells were analyzed by flow cytometry. **b** LPS-primed BMDMs were untreated or treated rhodamine-labeled SiO_2_ NPs and FITC-labeled TiO_2_ NPs (10 μg/cm^3^ each) for 30 min at 37 °C, and were stained with Hoechst 33482. After fixation with 4% paraformaldehyde, cells were analyzed by confocal microscopy. *White thick bars* indicate 10 μm. A higher magnification of the *white square* in the middle image is shown in the rightmost image. **c**, **d** LPS-primed BMDMs were stained with LysoTracker-Red and were untreated or stimulated with FITC-labeled SiO_2_ NPs or FITC-labeled TiO_2_ NPs (10 μg/cm^3^ each) for 30 min at 37 °C. After fixation with 4% paraformaldehyde, cells were analyzed by confocal microscopy. *White thick bars* indicate 10 μm in (**c**). FITC and LysoTracker signal intensities in area indicated by *white thin arrows* were measured and shown in (**d**). **e** LPS-primed BMDMs were untreated (*white bars*) or pretreated with YVAD-CHO (20 μM: *black bars*), Bafilomycin A1 (250 nM: *gray bars*), or BHA (100 μM: *red bars*) for 1 h at 37 °C, and then BMDMs were stimulated with SiO_2_ NPs and TiO_2_ NPs (10 μg/cm^3^ each) or ATP (1 mM) for 4 h at 37 °C. The amount of IL-1β in culture supernatants was measured by ELISA. Data are indicated as the mean + S.D. **f** LPS-primed BMDMs were untreated (*white bars*) or pretreated with YVAD-CHO (20 μM: *black bars*) or BHA (100 μM: *red bars*) for 1 h at 37 °C, and then were treated with DHR123 (1 μM) and NPs (10 μg/cm^3^ each) for 4 h at 37 °C. Percent DHR123-positive cells in propidium iodide-negative cells (Percent ROS-producing cells in live cells) was calculated by flow cytometry. Data are indicated as the mean + S.D. Similar results were obtained in at least three independent experiments

To address the mechanism underlying the synergistic induction of inflammation by SiO_2_ and TiO_2_ NPs, we first examined whether a single macrophage simultaneously recognizes both SiO_2_ and TiO_2_ NPs, or whether distinct macrophages recognize each NP in order to synergistically induce IL-1β secretion. To this end, we cultured BMDMs with rhodamine-labeled SiO_2_ NPs and/or FITC-labeled TiO_2_ NPs and analyzed these cells by flow cytometry. We observed that about 50% (SiO_2_ NPs) and 80% (TiO_2_ NPs) of the BMDM population recognized each NP (Fig. 2a). In BMDMs treated with both NPs, the SiO_2_ recognition level paralleled the TiO_2_ recognition level, suggesting that a single macrophage simultaneously recognize both NPs. Therefore, we hypothesized that SiO_2_ and TiO_2_ NPs may co-localize within macrophages. However, unexpectedly, confocal microscopy showed differential intracellular localization of rhodamine-SiO_2_ and FITC-TiO2 NPs in macrophages (Fig. 2b). To further analyze the intracellular localization of NPs, we cultured macrophages with FITC-SiO_2_ or FITC-TiO_2_ NPs and stained these cells with LysoTracker-Red. We observed that the fluorescence intensity of FITC-SiO_2_ NPs, but not of FITC-TiO_2_ NPs, merged with LysoTracker intensity (Fig. 2c, d), suggesting that SiO_2_ NPs, but not TiO_2_ NPs, localize within lysosomes.

### Involvement of lysosomal damage and ROS in the synergistic induction of IL-1β secretion by SiO_2_ and TiO_2_ NPs

We next addressed the involvement of lysosomal damage and ROS in SiO_2_ and TiO_2_ NP-induced synergistic IL-1β secretion. Consistent with lysosomal localization of SiO_2_ NPs (Fig. 2c, d), IL-1β secretion was completely blocked by bafilomycin A1, a specific inhibitor of vacuolar-type H^+^-ATPase that raises lysosomal pH and degrades the lysosomal proteinases [21] (Fig. 2e). In contrast, the blocking effect of an antioxidant, butylated hydroxyanisole (BHA), was only partial (Fig. 2e). On the other hand, neither bafilomycin A1 nor BHA affected ATP-induced IL-1β secretion (Fig. 2e). While involvement of lysosomal damage in particle-induced inflammasome activation has been widely accepted, the involvement of ROS in this process is highly controversial [22, 23]. Thus, we investigated ROS production and its inhibition by BHA in macrophages treated with NPs. Unexpectedly, 10 μg/cm^3^ of TiO_2_ NPs increased the population of DHR123-positive cells, indicating that TiO_2_ NPs produced a marked level of ROS (Fig. 2f), nevertheless IL-1β secretion was not induced (Fig. 1a). Moreover, there was no synergistic production of ROS in response to the mixture of SiO_2_ and TiO_2_ NPs. Further, ROS production was inhibited to a greater level by hydrophilic ascorbic acid than hydrophobic α-tocopherol (Additional file 1: Figure S4a), suggesting that ROS is produced in hydrophilic environments rather than in lipophilic environments. We next analyzed the oxidative damage by using Liperfluo, a new fluorescent probe, to detect lipid hydroperoxides [24] and by measuring the GSH/GSSG ratio. Although SiO_2_ and TiO_2_ NPs did not increase the population of Liperfluo-positive cells (Additional file 1: Figure S4b), these NPs reduced the GSH/GSSG ratio, an effect which was ameliorated by BHA or ascorbic acid (Additional file 1: Figure S4c). These results suggest that SiO_2_ and TiO_2_ NPs cause cytosolic oxidative damage. Of note, while BHA almost completely blocked ROS production (Fig. 2f) and oxidative stress (Additional file 1: Figure S4c), its effect on IL-1β secretion was only partial (Fig. 2e). Taken together, these results suggest that IL-1β secretion occurs following lysosomal damage and that while ROS alone does not trigger IL-1β secretion, it may enhance cellular stress leading to IL-1β secretion.

### A mixture of SiO_2_ and TiO_2_ NPs forms a colloidally stable complex in the presence of divalent cations

**Fig. 3:**
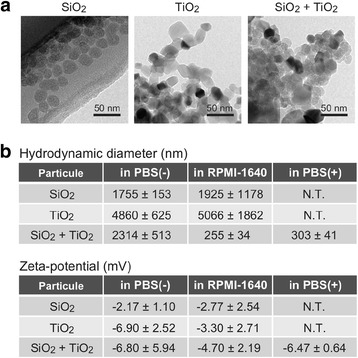
Characterization of SiO_2_ and TiO_2_ NP mixtures. **a** NPs were analyzed by transmission electron microscopy. **b** Hydrodynamic diameter and zeta-potential of NPs in PBS(−), PRMI-1640, or PBS(+) at pH7.2-7.4 were measured by dynamic light scattering. N.T.; not tested. Similar results were obtained in two independent experiments

To further address the mechanism underlying the synergistic induction of inflammation by SiO_2_ and TiO_2_ NPs, we characterized mixtures of these NPs. Transmission electron microscopy (TEM) images revealed that primary diameters of individual SiO_2_ and TiO_2_ NPs were clearly smaller than 50 nm (Fig. 3a). However, these hydrodynamic diameters were determined by dynamic light scattering and were of the micrometer scale (Fig. 3b). The estimated sizes were derived from the formation of aggregates among NPs, which formed immediately after sonication. In addition, the aggregate size was uncontrollable and increased over time. On the other hand, the mixture of SiO_2_ and TiO_2_ NPs was a relatively monodisperse complex with a size of ~250 nm in RPMI-1640 medium (Fig. 3b). Furthermore, the complex NPs showed colloid stability and the smaller aggregate size and dispersion was maintained even 24 h after sonication. Interestingly, complex nanoparticles were not detected in PBS(−). Therefore, we evaluated the mixture of these NPs in PBS(+) containing Mg^2+^ and Ca^2+^ (0.4 mM each) and observed similar complex formation. These results suggest that the mixture of SiO_2_ and TiO_2_ NPs forms a colloidally stable complex in the presence of divalent cations. While this precise mechanism remains unknown, divalent cations might stabilize the interaction between slightly negatively charged SiO_2_ and TiO_2_ NPs.

## Discussion

Given that a wide variety of ENMs are currently produced worldwide [1, 2], there is a significant risk for our bodies to be simultaneously exposed to several different types of these molecules. In this study, we provide evidence that SiO_2_ and TiO_2_ NPs, which are the most frequently used ENMs [2], synergistically induce IL-1β secretion in macrophages and subsequent pulmonary inflammation in mice at low concentrations/doses. The cellular mechanisms underlying this include SiO_2_ NP-induced lysosomal stress and TiO_2_-induced ROS production, which together synergistically induce macrophage cellular stress leading to inflammasome activation. This finding indicates that remarkably low doses of NPs may induce unexpected inflammation when our bodies are exposed to them in conjunction with other NPs.

We also provide evidence that individual SiO_2_ and TiO_2_ NPs easily aggregate to form micrometer sized particles, whereas in the presence of divalent cations mixtures of these NPs form colloidally stable complexes with sizes of ~250 nm. Because of body chemistry, it is likely this phenomenon occurs in humans when simultaneously exposed to both NPs. Given that smaller sized particles are more inflammatory [25, 26], this phenomenon may contribute to the synergistic inflammation caused by SiO_2_ and TiO_2_ NPs.

Particle-induced IL-1β secretion from macrophages is almost completely blocked by cytochalasin D, an inhibitor of actin polymerization and phagocytosis [9, 16, 17], suggesting that internalization of particles is required for IL-1β secretion. However, given that macrophages efficiently internalized even non-inflammatory NPs (data not shown), internalization of particles alone is not sufficient for the induction of IL-1β secretion. Numerous studies have shown that NLRP3-deficient macrophages do not induce IL-1β after internalization of particles, indicating that cytoplasmic NLRP3 inflammasome activation is indispensable for NP-induced IL-1β secretion [25]. Because NLRP3 is activated by various stresses, including bacterial toxins and cellular ion perturbation [13], it is unlikely that NLRP3 directly senses particulate substances. Rather, particle-induced lysosomal stress and/or ROS are considered likely mechanisms for activation of NLRP3. In this study, TiO_2_ but not SiO_2_ NPs induced high levels of ROS production; nevertheless, TiO_2_ NPs alone did not induce IL-1β, suggesting that ROS is not sufficient to activate inflammasomes. Rather, TiO_2_-induced ROS production may enhance SiO_2_-induced cellular stress leading to inflammasome activation.

Particle-induced ROS production depends on particle characteristics and cell types. For instance, TiO_2_ but not SiO_2_ NPs produce ROS in dendritic cells [27], which is consistent with our study. On the other hand, silica crystals produce ROS in macrophages [15]. In colon carcinoma cell lines and a keratinocyte cell line, SiO_2_ NPs as well as TiO_2_ NPs produce ROS [28, 29]. While the molecular mechanisms by which particles produce ROS remain unknown, it is considered that, in addition to the intrinsic ROS production by particles themselves, the NADPH oxidase pathway and the damage to mitochondria leads to intracellular ROS production [26], possibly in a cell-type specific manner.

In addition to the mechanisms underlying ROS production, it also remains unknown how particles cause lysosomal stress and why SiO_2_ is the only particulate that strongly induces stress capable of causing NLRP3 inflammasome activation. Moreover, further studies will be required to address whether a colloidally stable complex of SiO_2_ and TiO_2_ NPs enhances cellular stress.

For the risk assessment of NPs, it is important to use their optimal doses. The dose used in this study [5 mg/kg (~100 μg/head)] is largely equivalent to 9 working days at the Danish occupational exposure level according to previous studies [30, 31]. On the other hand, Oberdorster et al. [32] proposed that intratracheal instillations of several hundred μg into a rat do not resemble a relevant in vivo inhalation exposure because this dose is too high. However, the use of doses that closely reflect the expected exposure levels may also lead to misleading risk assessment because experimental rodents live in abnormally hygienic facilities. In contrast, humans are exposed to various microbes and environmental pollutants daily. Notably, Beura et al. [33] has recently reported that laboratory mice do not reflect relevant aspects of human immune systems, and proposed that mice exposed to physiological microbes have more similar immune systems to adult humans. Given that NPs activate the immune system, further studies will be required to determine whether physiological microbes impact NP toxicity.

## Conclusions

In this study, we provide evidence that SiO_2_ and TiO_2_ NPs synergistically induce macrophage inflammatory responses and subsequent lung inflammation at doses/concentrations where individual NPs do not induce inflammation. While there are numerous reports that address the toxicity of single ENMs, the synergistic toxicity of multiple ENMs is poorly understood. Given that our bodies are at risk of being simultaneously exposed to various ENMs and that environmental particulate matter contains various substances including SiO_2_, assessment of the synergistic toxicity of multiple particles is of paramount importance.

## Methods

### Particles

SiO_2_ NPs (Product # 43-00-301, Lot # 1801243–02, 2591343–03) and rhodamine-labeled SiO_2_ NPs (Product # 40-00-301, Lot # 0801140–04) (both amorphous structure, primary diameter 30 nm) were purchased from Micromod Partikeltechnologie GmbH (Rostock, Germany). According to this manufacturer’s information, SiO_2_ NPs were prepared by a modified Stoeber process [34] although the detail protocol is confidential. TiO_2_ NPs (anatase:rutile = 80:20, primary diameter 21 nm) were purchased from Sigma-Aldrich (St Louis, MO). TiO_2_ NPs were labeled with FITC as described previously [35] with minor modifications. In brief, 10 mM dopamine (Sigma-Aldrich) and 10 mM FITC (Sigma-Aldrich) were mixed in 50 mM Tris–HCl (pH 9.0) and incubated for 3 h at room temperature, and the resulting FITC-dopamine was mixed with nano-TiO_2_ crystal dispersion in distilled water for 30 min at 37 °C. Free FITC-dopamine was removed by extensive washing with 5% FBS/PBS. The fluorescence spectra of FITC-modified TiO_2_ NPs was measured (Additional file 1: Figure S5). All particles were dispersed by bath sonicator before addition to cells.

### Characterization of NPs

The primary size and shape of SiO_2_ and TiO_2_ NPs were analyzed by using an JEM-2100 F electron microscope (JEOL, Tokyo, Japan) with a 200 kV accelerating voltage. The hydrodynamic diameter and zeta-potential of NPs were measured in PBS(−), RPMI-1640 medium (Sigma-Aldrich), or DPBS(+) (pH7.2-7.4) (Thermo Fisher Scientific, Waltham, MA) at 37 °C by nano Partica SZ-1000 (HORIBA, Kyoto, Japan).

### Mice

C57BL/6 N female mice (5–6 weeks old) were purchased from Japan CLEA (Tokyo, Japan). Mice were maintained under specific pathogen-free conditions. All mouse studies were performed according to the protocols approved by the Institutional Committee for Use and Care of Laboratory Animals of Tohoku University, which was granted by Tohoku University Ethics Review Board (No. 2014IrA-009).

### BMDMs

C57BL/6 N mouse BMDMs were grown in complete RPMI-1640 (RPMI-1640 supplemented with 10% fetal bovine serum, 100 U/ml penicillin, 100 μg/ml streptomycin, and 2 mM glutamine) containing 50 ng/ml of recombinant human macrophage colony-stimulating factor (rhM-CSF; Peprotech Inc., Rocky Hill, NJ) for 5 or 6 days.

### IL-1β secretion from BMDMs

BMDMs (1×10^5^/well) were seeded into 48-well plates (growth area per well: 0.75 cm^2^) and cultured overnight. Cells were then primed with 5 ng/ml of ultra-pure LPS (List Biological Laboratories, Campbell, CA). After 4 h, cells were stimulated with the indicated NPs or with 1 mM ATP (Wako, Osaka, Japan) for 4 h at 37 °C. For some experiments, LPS-primed BMDMs were pretreated with 20 μM of YVAD-CHO (Peptide Inst., Osaka, Japan), 250 nM of Bafilomycin A1 (Sigma-Aldrich), or 100 μM of BHA (Sigma-Aldrich) for 1 h, and were then stimulated with silica particles for 4 h. The amount of IL-1β in cell culture supernatants was measured using ELISA (R&D Systems, Minneapolis, MN) according to the manufacturer’s instructions.

### Inflammasome activation

Immunoblot analysis of inflammasome activation was performed as described previously [9]. Briefly, BMDMs (1×10^6^/well) were seeded in six-well plates (growth area per well: 9.6 cm^2^) and cultured overnight. Cells were primed with LPS (5 ng/ml) for 4 h at 37 °C. After replacing the media with serum-free media containing rhM-CSF (50 ng/ml), cells were stimulated with SiO_2_ NPs and/or TiO_2_ NPs (10 μg/cm^3^ each) for 2 h at 37 °C. Culture supernatants and total cell lysates were pooled and then clarified by centrifugation. Proteins were precipitated with Strataclean Resin (Stratagene, La Jolla, CA) and detected with anti-mouse IL-1β Ab (R&D systems) and anti-mouse caspase-1 Ab (Santa Cruz Biotech, Dallas, TX).

### Mouse model of lung inflammation

SiO_2_ NPs and/or TiO_2_ NPs [5 mg/kg (~100 μg/head) each] in PBS or the vehicle alone were injected i.t. into C57BL/6 N female mice (6–7 weeks old). According to previous studies [30, 31], this dose is largely equivalent to 9 working days at the Danish occupational exposure level for TiO_2_ (6.0 mg Ti/m^3^ ~ 9.75 mg TiO_2_/m^3^) (assuming 9% of particles reach the lung [36], 1.8 l/h inhaled air [37], and 8 h working days). Twenty-four h after injection, lung inflammation was analyzed by micro-CT scan using a LaThetaTM LCT-200 (Hitachi-ALOKA, Tokyo, Japan). BALF was then harvested from mice, and the BALF cells were counted and stained with APC-anti-Gr-1 mAb (BioLegend, San Diego, CA). Cells were analyzed using an Accuri C6 flow cytometer (BD Biosciences, San Jose, CA).

### Recognition of NPs by BMDMs

Rhodamine-labeled SiO_2_ NPs and/or FITC-labeled TiO_2_ NPs (10 μg/cm^3^ each) were added to LPS-primed BMDMs (1×10^5^/well) seeded into 48-well plates (growth area per well: 0.75 cm^2^) for 30 min at 37 °C. After washing twice with PBS, cells were harvested by trypsinization. Recognition of NPs by BMDMs was analyzed by Accuri C6 flow cytometry.

### Intracellular localization of NPs

For SiO_2_ and TiO_2_ NP localization, BMDMs (1×10^5^/well) grown on poly-L-lysine (Wako)-coated glass coverslips were cultured with rhodamine-labeled SiO_2_ NPs and FITC-labeled TiO_2_ NPs (10 μg/cm^3^ each) in 24-well plates (growth area per well: 2.0 cm^2^) for 30 min at 37 °C, then washed with PBS and stained with Hoechst33342 (Dojindo, Kumamoto, Japan) to visualize nuclei. After fixation with 4% formaldehyde solution, cells were analyzed using an LSM800 confocal laser microscope (Zeiss, Oberkochen, Germany) equipped with a 40x objective lens. As for lysosomal localization analysis, BMDMs grown on poly-L-lysine-coated glass coverslips were stained with LysoTracker-Red DBD99 (75 nM; Invitrogen) for 1 h at 37 °C, and were cultured with FITC-labeled SiO_2_ NPs or FITC-labeled TiO_2_ NPs (10 μg/cm^3^ each) for 30 min at 37 °C. After washing with PBS and fixation with 4% formaldehyde solution, cells were analyzed using confocal microscopy as above.

### ROS production in macrophages

LPS-primed BMDMs were treated with dihydrorhodamine (DHR) 123 (1 μM; Cayman Chemical, Ann Arbor, MI) and SiO_2_ NPs and/or TiO_2_ NPs (10 μg/cm^3^ each) in 48-well plates (growth area per well: 0.75 cm^2^) for 4 h. Cells were harvested and stained with propidium iodide (PI, 0.5 μg/ml; Sigma-Aldrich). The percent of DHR123-positive cells in PI-negative cells was measured using an Accuri C6 flow cytometer.

### Statistical analyses

Unpaired two-tailed Student’s *t*-test was performed to analyze differences between two groups; one-way ANOVA with Holm’s post hoc test was performed for multiple groups. *P* < 0.05 was considered to be statistically significant. All data are represented as the mean + S.D.

## Additional file

Additional file 1: Figure S1. IL-1β secretion from bone marrow-derived macrophages (BMDMs) stimulated with various inorganic nanoparticles (NPs). **a** LPS-primed (black circles) or unprimed (white circles) BMDMs were stimulated with the indicated dose of NPs for 4 h at 37 °C. The amount of IL-1β in culture supernatants was measured by ELISA. **b** LPS-primed BMDMs were stimulated with the indicated combination of NPs (10 μg/cm^3^ each) for 4 h at 37 °C. The amount of IL-1β in culture supernatants was measured by ELISA. S.D. was less than 10% of the mean of triplicates (not shown). N.D.; not detected. Similar results were obtained in three independent experiments. **Figure S2.** Concentration-dependent IL-1β secretion from BMDMs stimulated with SiO_2_ and/or TiO_2_ NPs. LPS-primed BMDMs were stimulated with the indicated concentration of SiO_2_ and/or TiO_2_ NPs for 4 h at 37 °C. The amount of IL-1β in culture supernatants was measured by ELISA. Data are shown as mean + S.D. N.D.; not detected. **P* < 0.05, ***P* < 0.01, compared to other cells treated with the same concentration of SiO_2_ NPs, Holm’s *post hoc* test. Similar results were obtained in two independent experiments. **Figure S3.** Dose-dependent lung inflammation in mice treated with SiO_2_ and/or TiO_2_ NPs. C57BL/6 mice were intratracheally treated with PBS alone or with the indicated dose of SiO_2_ and/or TiO_2_ NPs (*N* = 3 per group). Twenty-four h after injection, lung inflammation was analyzed by micro-computed tomography in **a**. Bronchoalveolar lavage fluid (BALF) was harvested from these mice, and the total cell number in BALF was counted. Then cells were stained with fluorescently-labeled anti-Gr-1 mAb and analyzed by flow cytometry. Gr-1-positive cell number in BALF was calculated and is shown as the mean + S.D. in **b**. **P* < 0.05 compared to others treated with the same dose of NPs, Holm’s *post hoc* test. Similar results were obtained in two independent experiments. **Figure S4.** Oxidative stress in BMDMs treated with SiO_2_ and TiO_2_ NPs. **a** LPS-primed BMDMs were untreated or pretreated with the indicated antioxidant (100 μM each) for 1 h at 37 °C, and then were treated with or without SiO_2_ and TiO_2_ NPs (10 μg/cm^3^ each) for 4 h at 37 °C in the presence of DHR123 (1 μM). Percent DHR123-positive cells (Percent ROS-producing cells) was calculated by flow cytometry. **b** LPS-primed BMDMs were stimulated as described in **a** in the presence of Liperfluo (20 μM). Percent Liperfluo-positive cells (Percent lipid hydroperoxide-positive cells) was calculated by flow cytometry. **c** LPS-primed BMDMs were stimulated as described in **a**. The intracellular GSH and GSSG levels were determined, and the GSH/GSSG ratios were calculated. Data are indicated as the mean + S.D. Similar results were obtained in three (**a**, **b**) or two (**c**) independent experiments. n.s., not significant. ***P* < 0.01, two-tailed Student’s *t*-test. **Figure S5.** Fluorescence spectra of FITC-modified TiO2 NPs dispersed in PBS(-), pH7.4. (ZIP 1154 kb)

## Abbreviations

BALF: Bronchoalveolar lavage fluid
BHA: Hydroxyanisole
BMDMs: Bone marrow-derived macrophages
ENMs: Engineered nanomaterials
LPS: Lipopolysaccharide
NLRP3: Nod-like receptor protein 3
NPs: Nanoparticles
PAMPs: Pathogen-associated molecular patterns
ROS: Reactive oxygen species
