# Supplementary material for: SiO2 and TiO2 nanoparticles synergistically trigger macrophage inflammatory responses
Source: Part Fibre Toxicol. 2017 Apr 11;14:11. doi: 10.1186/s12989-017-0192-6 (PMC5387387; doi:10.1186/s12989-017-0192-6)
Supplement: Additional file 1: Figure S1. — IL-1β secretion from bone marrow-derived macrophages (BMDMs) stimulated with various inorganic nanoparticles (NPs). a LPS-primed (black circles) or unprimed (white circles) BMDMs were stimulated with the indicated dose of NPs for 4 h at 37 °C. The amount of IL-1β in culture supernatants was measured by ELISA. b LPS-primed BMDMs were stimulated with the indicated combination of NPs (10 μg/cm3 each) for 4 h at 37 °C. The amount of IL-1β in culture supernatants was measured by ELISA. S.D. was less than 10% of the mean of triplicates (not shown). N.D.; not detected. Similar results were obtained in three independent experiments. Figure S2. Concentration-dependent IL-1β secretion from BMDMs stimulated with SiO2 and/or TiO2 NPs. LPS-primed BMDMs were stimulated with the indicated concentration of SiO2 and/or TiO2 NPs for 4 h at 37 °C. The amount of IL-1β in culture supernatants was measured by ELISA. Data are shown as mean + S.D. N.D.; not detected. *P < 0.05, **P < 0.01, compared to other cells treated with the same concentration of SiO2 NPs, Holm’s post hoc test. Similar results were obtained in two independent experiments. Figure S3. Dose-dependent lung inflammation in mice treated with SiO2 and/or TiO2 NPs. C57BL/6 mice were intratracheally treated with PBS alone or with the indicated dose of SiO2 and/or TiO2 NPs (N = 3 per group). Twenty-four h after injection, lung inflammation was analyzed by micro-computed tomography in a. Bronchoalveolar lavage fluid (BALF) was harvested from these mice, and the total cell number in BALF was counted. Then cells were stained with fluorescently-labeled anti-Gr-1 mAb and analyzed by flow cytometry. Gr-1-positive cell number in BALF was calculated and is shown as the mean + S.D. in b. *P < 0.05 compared to others treated with the same dose of NPs, Holm’s post hoc test. Similar results were obtained in two independent experiments. Figure S4. Oxidative stress in BMDMs treated with SiO2 and TiO2 NPs. a LPS-primed BMDMs were untreated or [file 12989_2017_192_MOESM1_ESM.zip › Supplemental Text-5.docx]

**Supplemental Figure legends**

**Fig. S1** IL-1β secretion from bone marrow-derived macrophages (BMDMs) stimulated with various inorganic nanoparticles (NPs). **a** LPS-primed (black circles) or unprimed (white circles) BMDMs were stimulated with the indicated dose of NPs for 4 h at 37°C. The amount of IL-1β in culture supernatants was measured by ELISA. **b** LPS-primed BMDMs were stimulated with the indicated combination of NPs (10 μg/cm^3^ each) for 4 h at 37°C. The amount of IL-1β in culture supernatants was measured by ELISA. S.D. was less than 10% of the mean of triplicates (not shown). N.D.; not detected. Similar results were obtained in three independent experiments.

**Fig. S2** Concentration-dependent IL-1β secretion from BMDMs stimulated with SiO_2_ and/or TiO_2_ NPs. LPS-primed BMDMs were stimulated with the indicated dose of SiO_2_ and/or TiO_2_ NPs for 4 h at 37°C. The amount of IL-1β in culture supernatants was measured by ELISA. Data are shown as mean + S.D. N.D.; not detected. **P*<0.05, ***P*<0.01, to the others treated with the same concentration of SiO_2_ NPs, Holm’s *post hoc* test. Similar results were obtained in two independent experiments.

**Fig. S3** Dose-dependent lung inflammation in mice treated with SiO_2_ and/or TiO_2_ NPs. C57BL/6 mice were intratracheally treated with PBS alone or with the indicated dose of SiO_2_ and/or TiO_2_ NPs (N=3 per group). Twenty-four h after injection, lung inflammation was analyzed by micro-computed tomography in **a**. Bronchoalveolar lavage fluid (BALF) was harvested from these mice, and total cell number in BALF was counted. Then cells were stained with fluorescently-labeled anti-Gr-1 mAb and analyzed by flow cytometry. Gr-1-positive cell number in BALF was calculated and indicated as the mean + S.D. in **b**. **P*<0.05 to the others treated with the same dose of NPs, Holm’s *post hoc* test. Similar results were obtained in two independent experiments.

**Fig. S4** Oxidative stress in BMDMs treated with SiO_2_ and TiO_2_ NPs. **a** LPS-primed BMDMs were untreated or pretreated with the indicated antioxidant (100 μM each) for 1 h at 37°C, and then were treated with or without SiO_2_ and TiO_2_ NPs (10 μg/cm^3^ each) for 4 h at 37°C in the presence of DHR123 (1 μM). Percent DHR123-positive cells (Percent ROS-producing cells) was calculated by flow cytometry. **b** LPS-primed BMDMs were stimulated as described in **a** in the presence of Liperfluo (20 μM). Percent Liperfluo-positive cells (Percent lipid hydroperoxide-positive cells) was calculated by flow cytometry. **c** LPS-primed BMDMs were stimulated as described in **a**. The intracellular GSH and GSSG levels were determined, and the GSH/GSSG ratios were calculated. Data are indicated as the mean + S.D. Similar results were obtained in three (**a**, **b**) or two (**c**) independent experiments. n.s., not significant. ***P*<0.01, two-tailed Student’s t-test.

**Fig. S5** Fluorescence spectra of FITC modified TiO2 NPs dispersed in PBS(-), pH7.4.

**Supplemental Methods**

**Particles**

NiO NPs (primary diameter <50 nm), Al_2_O_3_ NPs (primary diameter 30-60 nm), ZnO NPs (primary diameter <50 nm), and Ag NPs (primary diameter 40 nm) were all purchased from Sigma-Aldrich (St Louis, MO).

**Lipid hydroperoxides in macrophages**

LPS-primed BMDMs were treated simultaneously with Liperfluo (20 μM; Dojindo, Kumamoto, Japan), SiO_2_ and TiO_2_ NPs (10 μg/cm^3^ each) for 4 h. In some groups, cells were pretreated with BHA (100 μM, Sigma-Aldrich), ascorbic acid (100 μM, Wako, Osaka, Japan) or α-tocopherol (100 μM, Sigma-Aldrich) 1 hr before NP stimulation. The percent of Liperfluo-positive cells was measured using an Accuri C6 flow cytometer (BD Biosciences, San Jose, CA).

**Determination of cellular GSH/GSSG ratios**

LPS-primed BMDMs were treated simultaneously with or without SiO_2_ and TiO_2_ NPs (10 μg/cm^3^ each) for 4 h. In some groups, cells were pretreated with BHA, ascorbic acid, or α-tocopherol (100 μM each) 1 hr before NP stimulation. The cellular GSH and GSSG levels, and the GSH/GSSG ratios were determined by GSSG/GSH quantification kit (Dojindo) according to the manufacturer’s instruction.

**Fluorescence spectra of FITC on NPs**

Fluorescence spectra of FITC-modified TiO_2_ NPs were recorded with a fluorescence spectrometer (F-2500, Hitachi, Tokyo, Japan). The fluorescence spectra of FITC dye labeled on TiO_2_ NPs was dispersed in PBS(-) and measured with the concentration ranged from 0.0078 μg/cm^3^ to 0.5 μg/cm^3^ (Excitation wavelength = 488 nm).
