# Supplementary figures and images for: SiO2 and TiO2 nanoparticles synergistically trigger macrophage inflammatory responses
Source: Part Fibre Toxicol. 2017 Apr 11;14:11. doi: 10.1186/s12989-017-0192-6 (PMC5387387; doi:10.1186/s12989-017-0192-6)

Fig. S5

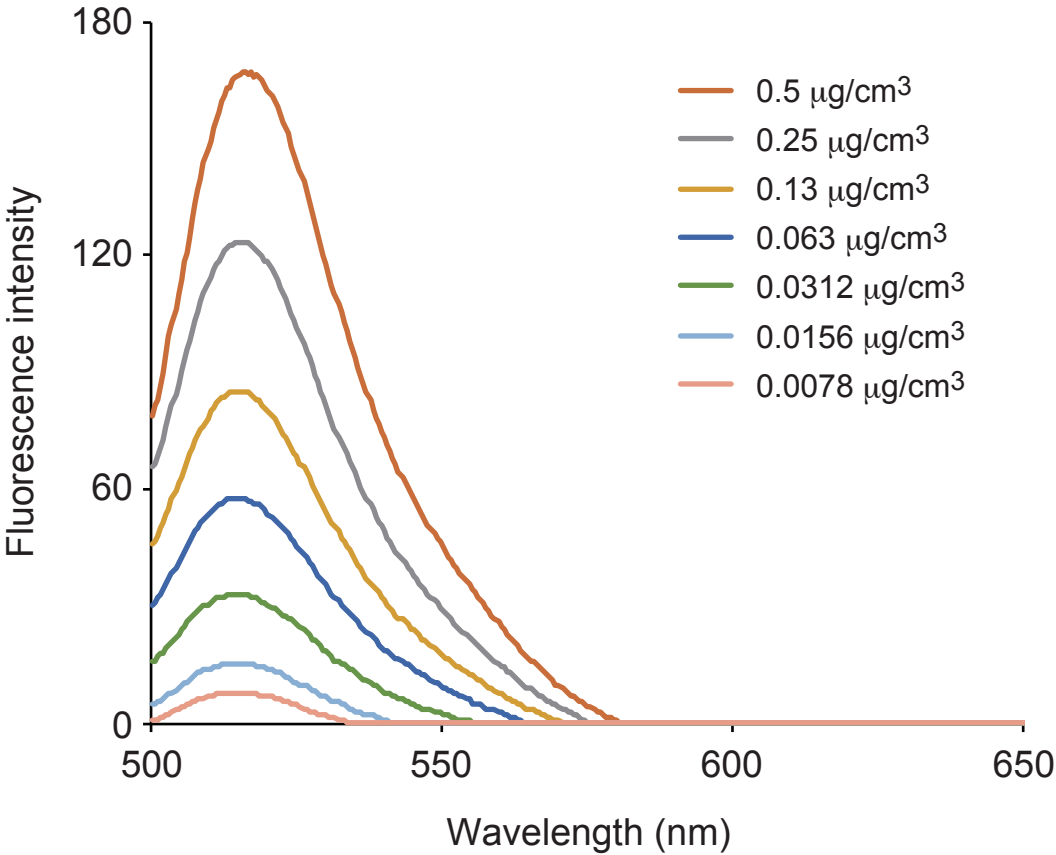

Supplement: Additional file 1: Figure S1. — IL-1β secretion from bone marrow-derived macrophages (BMDMs) stimulated with various inorganic nanoparticles (NPs). a LPS-primed (black circles) or unprimed (white circles) BMDMs were stimulated with the indicated dose of NPs for 4 h at 37 °C. The amount of IL-1β in culture supernatants was measured by ELISA. b LPS-primed BMDMs were stimulated with the indicated combination of NPs (10 μg/cm3 each) for 4 h at 37 °C. The amount of IL-1β in culture supernatants was measured by ELISA. S.D. was less than 10% of the mean of triplicates (not shown). N.D.; not detected. Similar results were obtained in three independent experiments. Figure S2. Concentration-dependent IL-1β secretion from BMDMs stimulated with SiO2 and/or TiO2 NPs. LPS-primed BMDMs were stimulated with the indicated concentration of SiO2 and/or TiO2 NPs for 4 h at 37 °C. The amount of IL-1β in culture supernatants was measured by ELISA. Data are shown as mean + S.D. N.D.; not detected. *P < 0.05, **P < 0.01, compared to other cells treated with the same concentration of SiO2 NPs, Holm’s post hoc test. Similar results were obtained in two independent experiments. Figure S3. Dose-dependent lung inflammation in mice treated with SiO2 and/or TiO2 NPs. C57BL/6 mice were intratracheally treated with PBS alone or with the indicated dose of SiO2 and/or TiO2 NPs (N = 3 per group). Twenty-four h after injection, lung inflammation was analyzed by micro-computed tomography in a. Bronchoalveolar lavage fluid (BALF) was harvested from these mice, and the total cell number in BALF was counted. Then cells were stained with fluorescently-labeled anti-Gr-1 mAb and analyzed by flow cytometry. Gr-1-positive cell number in BALF was calculated and is shown as the mean + S.D. in b. *P < 0.05 compared to others treated with the same dose of NPs, Holm’s post hoc test. Similar results were obtained in two independent experiments. Figure S4. Oxidative stress in BMDMs treated with SiO2 and TiO2 NPs. a LPS-primed BMDMs were untreated or [file 12989_2017_192_MOESM1_ESM.zip › Fig. S5.pdf]

Fig. S3

a

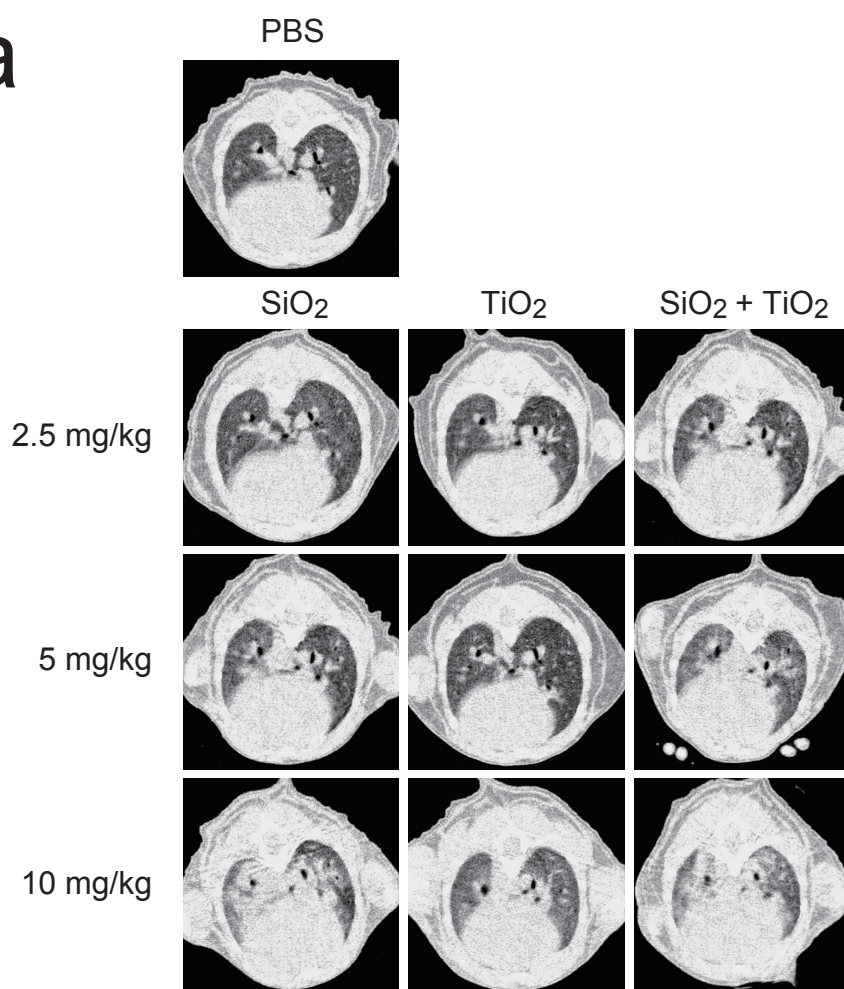

b

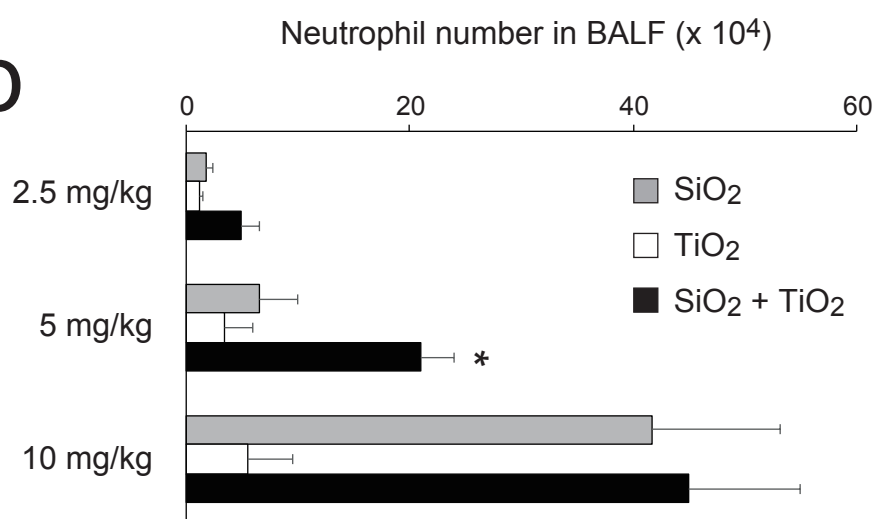

Supplement: Additional file 1: Figure S1. — IL-1β secretion from bone marrow-derived macrophages (BMDMs) stimulated with various inorganic nanoparticles (NPs). a LPS-primed (black circles) or unprimed (white circles) BMDMs were stimulated with the indicated dose of NPs for 4 h at 37 °C. The amount of IL-1β in culture supernatants was measured by ELISA. b LPS-primed BMDMs were stimulated with the indicated combination of NPs (10 μg/cm3 each) for 4 h at 37 °C. The amount of IL-1β in culture supernatants was measured by ELISA. S.D. was less than 10% of the mean of triplicates (not shown). N.D.; not detected. Similar results were obtained in three independent experiments. Figure S2. Concentration-dependent IL-1β secretion from BMDMs stimulated with SiO2 and/or TiO2 NPs. LPS-primed BMDMs were stimulated with the indicated concentration of SiO2 and/or TiO2 NPs for 4 h at 37 °C. The amount of IL-1β in culture supernatants was measured by ELISA. Data are shown as mean + S.D. N.D.; not detected. *P < 0.05, **P < 0.01, compared to other cells treated with the same concentration of SiO2 NPs, Holm’s post hoc test. Similar results were obtained in two independent experiments. Figure S3. Dose-dependent lung inflammation in mice treated with SiO2 and/or TiO2 NPs. C57BL/6 mice were intratracheally treated with PBS alone or with the indicated dose of SiO2 and/or TiO2 NPs (N = 3 per group). Twenty-four h after injection, lung inflammation was analyzed by micro-computed tomography in a. Bronchoalveolar lavage fluid (BALF) was harvested from these mice, and the total cell number in BALF was counted. Then cells were stained with fluorescently-labeled anti-Gr-1 mAb and analyzed by flow cytometry. Gr-1-positive cell number in BALF was calculated and is shown as the mean + S.D. in b. *P < 0.05 compared to others treated with the same dose of NPs, Holm’s post hoc test. Similar results were obtained in two independent experiments. Figure S4. Oxidative stress in BMDMs treated with SiO2 and TiO2 NPs. a LPS-primed BMDMs were untreated or [file 12989_2017_192_MOESM1_ESM.zip › Fig. S3-2.pdf]

Fig. S4

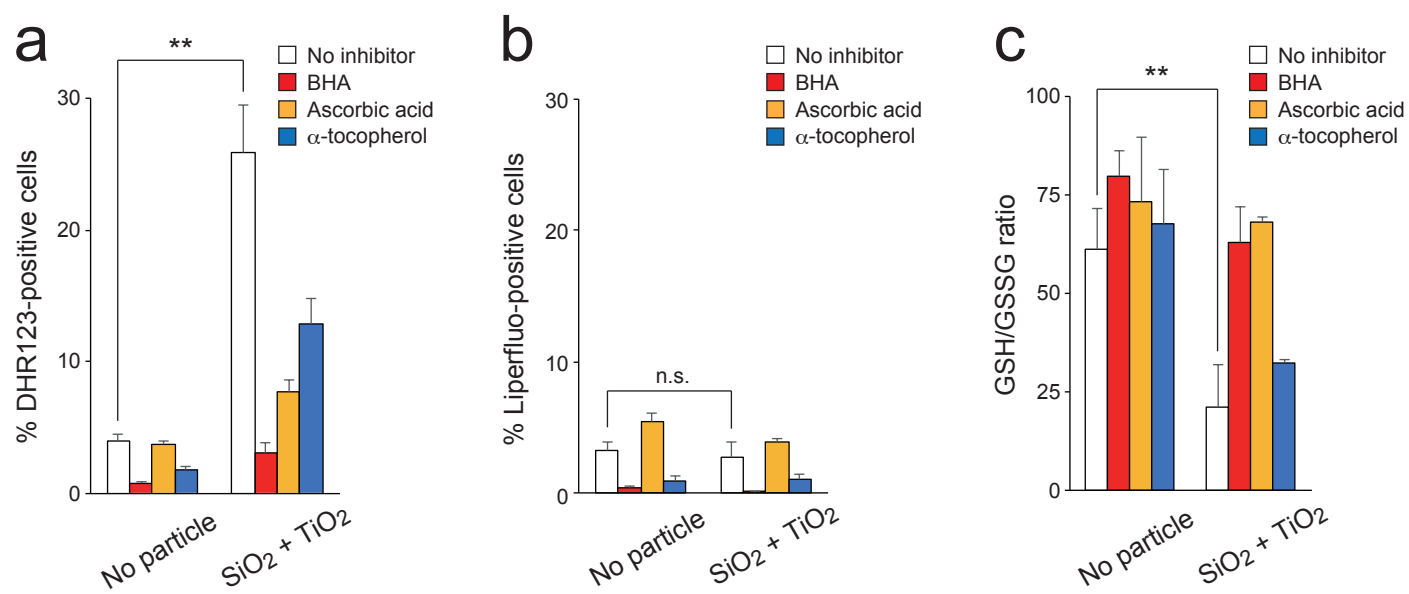

Supplement: Additional file 1: Figure S1. — IL-1β secretion from bone marrow-derived macrophages (BMDMs) stimulated with various inorganic nanoparticles (NPs). a LPS-primed (black circles) or unprimed (white circles) BMDMs were stimulated with the indicated dose of NPs for 4 h at 37 °C. The amount of IL-1β in culture supernatants was measured by ELISA. b LPS-primed BMDMs were stimulated with the indicated combination of NPs (10 μg/cm3 each) for 4 h at 37 °C. The amount of IL-1β in culture supernatants was measured by ELISA. S.D. was less than 10% of the mean of triplicates (not shown). N.D.; not detected. Similar results were obtained in three independent experiments. Figure S2. Concentration-dependent IL-1β secretion from BMDMs stimulated with SiO2 and/or TiO2 NPs. LPS-primed BMDMs were stimulated with the indicated concentration of SiO2 and/or TiO2 NPs for 4 h at 37 °C. The amount of IL-1β in culture supernatants was measured by ELISA. Data are shown as mean + S.D. N.D.; not detected. *P < 0.05, **P < 0.01, compared to other cells treated with the same concentration of SiO2 NPs, Holm’s post hoc test. Similar results were obtained in two independent experiments. Figure S3. Dose-dependent lung inflammation in mice treated with SiO2 and/or TiO2 NPs. C57BL/6 mice were intratracheally treated with PBS alone or with the indicated dose of SiO2 and/or TiO2 NPs (N = 3 per group). Twenty-four h after injection, lung inflammation was analyzed by micro-computed tomography in a. Bronchoalveolar lavage fluid (BALF) was harvested from these mice, and the total cell number in BALF was counted. Then cells were stained with fluorescently-labeled anti-Gr-1 mAb and analyzed by flow cytometry. Gr-1-positive cell number in BALF was calculated and is shown as the mean + S.D. in b. *P < 0.05 compared to others treated with the same dose of NPs, Holm’s post hoc test. Similar results were obtained in two independent experiments. Figure S4. Oxidative stress in BMDMs treated with SiO2 and TiO2 NPs. a LPS-primed BMDMs were untreated or [file 12989_2017_192_MOESM1_ESM.zip › Fig. S4.pdf]

Fig. S2

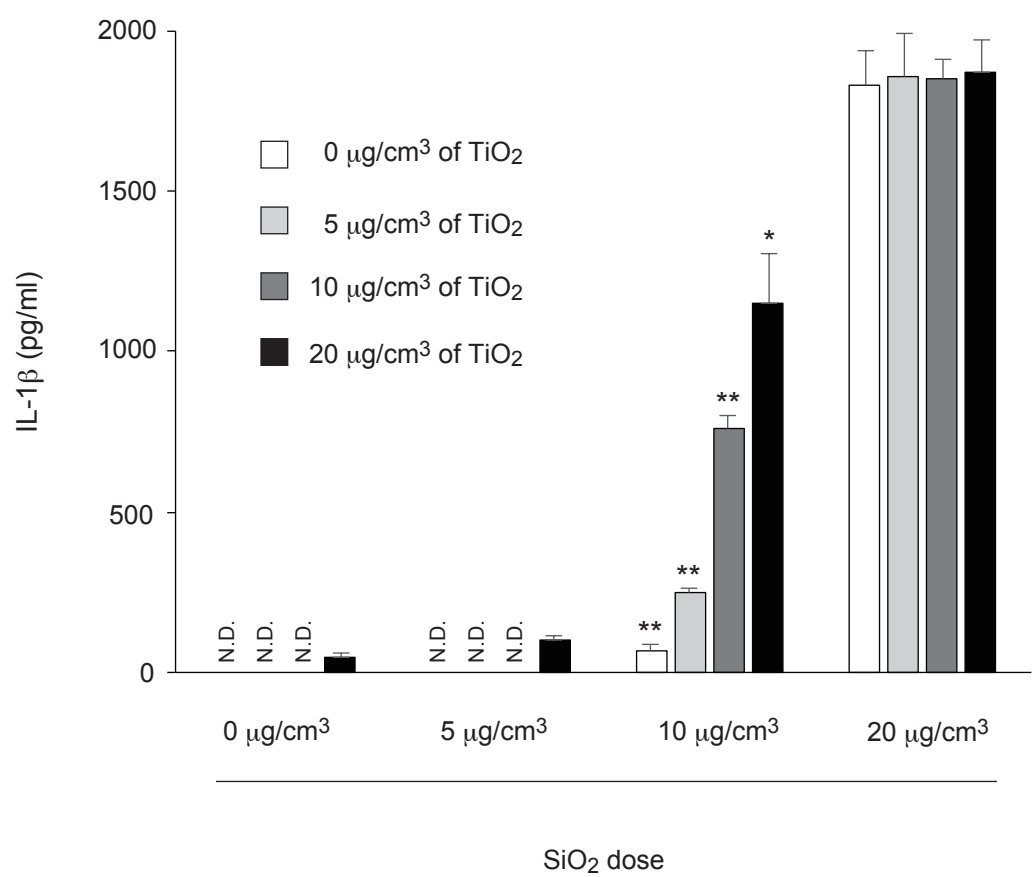

Supplement: Additional file 1: Figure S1. — IL-1β secretion from bone marrow-derived macrophages (BMDMs) stimulated with various inorganic nanoparticles (NPs). a LPS-primed (black circles) or unprimed (white circles) BMDMs were stimulated with the indicated dose of NPs for 4 h at 37 °C. The amount of IL-1β in culture supernatants was measured by ELISA. b LPS-primed BMDMs were stimulated with the indicated combination of NPs (10 μg/cm3 each) for 4 h at 37 °C. The amount of IL-1β in culture supernatants was measured by ELISA. S.D. was less than 10% of the mean of triplicates (not shown). N.D.; not detected. Similar results were obtained in three independent experiments. Figure S2. Concentration-dependent IL-1β secretion from BMDMs stimulated with SiO2 and/or TiO2 NPs. LPS-primed BMDMs were stimulated with the indicated concentration of SiO2 and/or TiO2 NPs for 4 h at 37 °C. The amount of IL-1β in culture supernatants was measured by ELISA. Data are shown as mean + S.D. N.D.; not detected. *P < 0.05, **P < 0.01, compared to other cells treated with the same concentration of SiO2 NPs, Holm’s post hoc test. Similar results were obtained in two independent experiments. Figure S3. Dose-dependent lung inflammation in mice treated with SiO2 and/or TiO2 NPs. C57BL/6 mice were intratracheally treated with PBS alone or with the indicated dose of SiO2 and/or TiO2 NPs (N = 3 per group). Twenty-four h after injection, lung inflammation was analyzed by micro-computed tomography in a. Bronchoalveolar lavage fluid (BALF) was harvested from these mice, and the total cell number in BALF was counted. Then cells were stained with fluorescently-labeled anti-Gr-1 mAb and analyzed by flow cytometry. Gr-1-positive cell number in BALF was calculated and is shown as the mean + S.D. in b. *P < 0.05 compared to others treated with the same dose of NPs, Holm’s post hoc test. Similar results were obtained in two independent experiments. Figure S4. Oxidative stress in BMDMs treated with SiO2 and TiO2 NPs. a LPS-primed BMDMs were untreated or [file 12989_2017_192_MOESM1_ESM.zip › Fig. S2-1.pdf]

Fig. S1

a

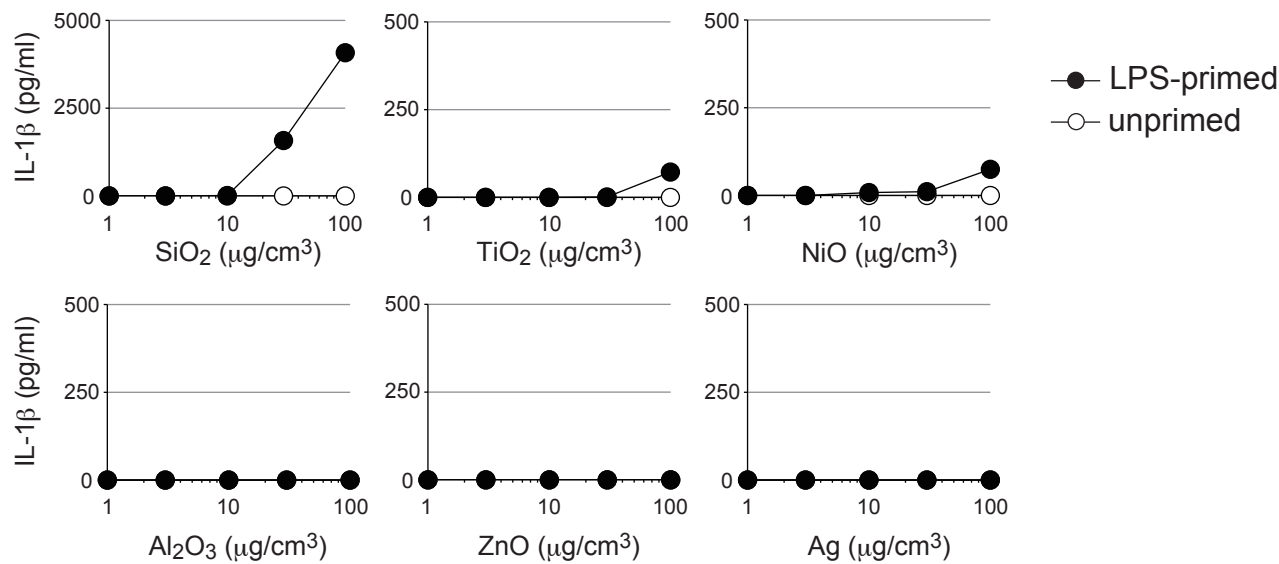

b

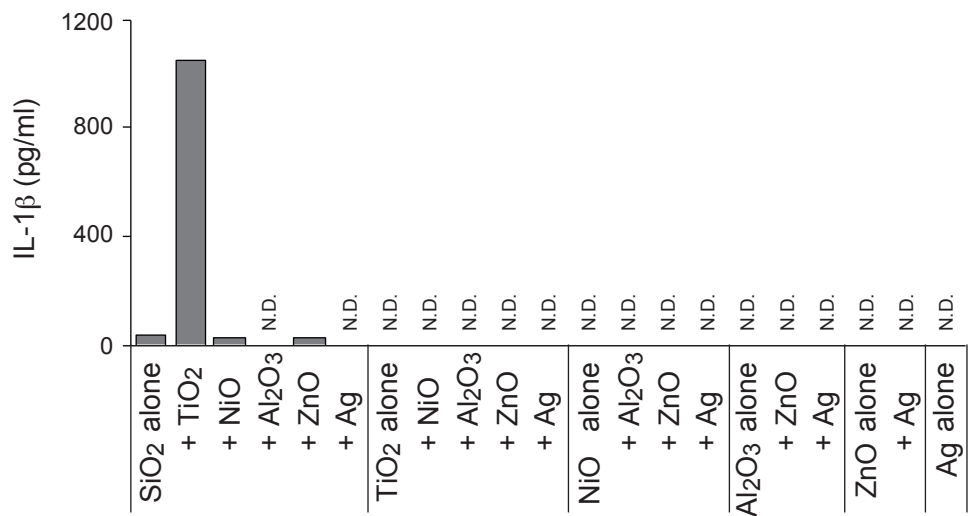

Supplement: Additional file 1: Figure S1. — IL-1β secretion from bone marrow-derived macrophages (BMDMs) stimulated with various inorganic nanoparticles (NPs). a LPS-primed (black circles) or unprimed (white circles) BMDMs were stimulated with the indicated dose of NPs for 4 h at 37 °C. The amount of IL-1β in culture supernatants was measured by ELISA. b LPS-primed BMDMs were stimulated with the indicated combination of NPs (10 μg/cm3 each) for 4 h at 37 °C. The amount of IL-1β in culture supernatants was measured by ELISA. S.D. was less than 10% of the mean of triplicates (not shown). N.D.; not detected. Similar results were obtained in three independent experiments. Figure S2. Concentration-dependent IL-1β secretion from BMDMs stimulated with SiO2 and/or TiO2 NPs. LPS-primed BMDMs were stimulated with the indicated concentration of SiO2 and/or TiO2 NPs for 4 h at 37 °C. The amount of IL-1β in culture supernatants was measured by ELISA. Data are shown as mean + S.D. N.D.; not detected. *P < 0.05, **P < 0.01, compared to other cells treated with the same concentration of SiO2 NPs, Holm’s post hoc test. Similar results were obtained in two independent experiments. Figure S3. Dose-dependent lung inflammation in mice treated with SiO2 and/or TiO2 NPs. C57BL/6 mice were intratracheally treated with PBS alone or with the indicated dose of SiO2 and/or TiO2 NPs (N = 3 per group). Twenty-four h after injection, lung inflammation was analyzed by micro-computed tomography in a. Bronchoalveolar lavage fluid (BALF) was harvested from these mice, and the total cell number in BALF was counted. Then cells were stained with fluorescently-labeled anti-Gr-1 mAb and analyzed by flow cytometry. Gr-1-positive cell number in BALF was calculated and is shown as the mean + S.D. in b. *P < 0.05 compared to others treated with the same dose of NPs, Holm’s post hoc test. Similar results were obtained in two independent experiments. Figure S4. Oxidative stress in BMDMs treated with SiO2 and TiO2 NPs. a LPS-primed BMDMs were untreated or [file 12989_2017_192_MOESM1_ESM.zip › Fig. S1-3.pdf]
